# The Forest of Biologists – for biologists, for biology

**DOI:** 10.1242/dmm.050178

**Published:** 2023-03-13

**Authors:** O. Claire Moulton, Matthew Freeman

**Affiliations:** ^1^ The Company of Biologists, Bidder Building, Station Road, Histon, Cambridge CB24 9LF, UK; ^2^ Sir William Dunn School of Pathology, University of Oxford, Oxford OX1 3RE, UK

We are excited to announce an ambitious new biodiversity project – The Forest of Biologists – creating, restoring and preserving important woodland habitats in the UK. This will help to counteract nature loss and safeguard some of the most critically endangered ecosystems for future generations.

We want to integrate this new initiative into the core of what we do as a publisher. For the next two years, we will be planting a new tree for every Research Article and Review article that we publish. We will also be funding the restoration and preservation of ancient woodland – these are some of the rarest and most biodiverse habitats in the UK – and dedicating these woodland trees to our peer reviewers, who help us to preserve the integrity of the scientific record.

This important work will be funded by The Company of Biologists as a not-for-profit publisher and UK charity. Our focus on the creation, restoration and preservation of precious woodland habitats reflects widespread concern among biologists worldwide about climate change and a global decline in biodiversity. After nearly 100 years of publishing journals, facilitating scientific meetings and providing charitable grants to support our communities, we want to play our part in supporting biology too. In linking this initiative to our authors and peer reviewers, we want to acknowledge the extraordinary support we receive from the communities that embrace Disease Models & Mechanisms and its sister journals: Development, Journal of Cell Science, Journal of Experimental Biology and Biology Open.

As we aim to make a positive difference, it's important that we think carefully and that our actions are directed and evaluated by science. We've therefore chosen to work with the Woodland Trust (https://www.woodlandtrust.org.uk/), the UK's largest woodland conservation charity focusing on the role that trees and woods play in tackling the threats of climate change and nature loss. In their words, ‘the benefits of trees in the fight against climate change are now well understood. They lock up carbon, reduce pollution and flooding, and support people, wildlife and farming in adapting to the climate crisis.’


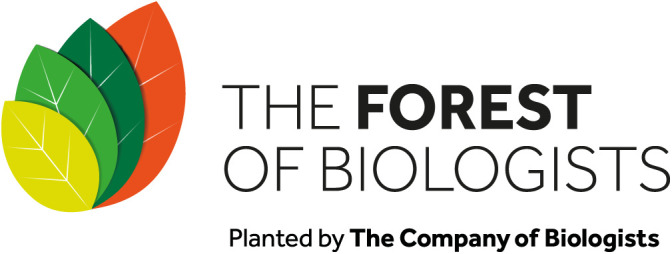


## Responsible planting of new trees

It's important to us as biologists that our trees are responsibly planted with long-term management and preservation in mind. Through our collaboration with the Woodland Trust, we're funding the planting of native trees in the Young People's Forest in Derbyshire (https://www.woodlandtrust.org.uk/visiting-woods/woods/young-peoples-forest-at-mead/). The grove will feature a range of native UK tree species including silver birch, oak, lime, alder, rowan and hawthorn – a good mix of species reduces the risks of vulnerability to diseases and pests. The site is rich in wildlife and, once complete, will include biodiverse ponds, open spaces and species-rich grassland.

*The nature and climate crises we face require urgent action – and that action needs to be directed and evaluated by science. That's why we are really excited to be working with The Company of Biologists on both the restoration of ancient woodlands and the creation of new woodlands. Ancient woodland is one of our most biodiverse habitats and using these as the building blocks for woodland and habitat expansion gives us the best opportunity to address these crises together*.

John Tucker, Woodland Trust Ambassador

**Figure:**
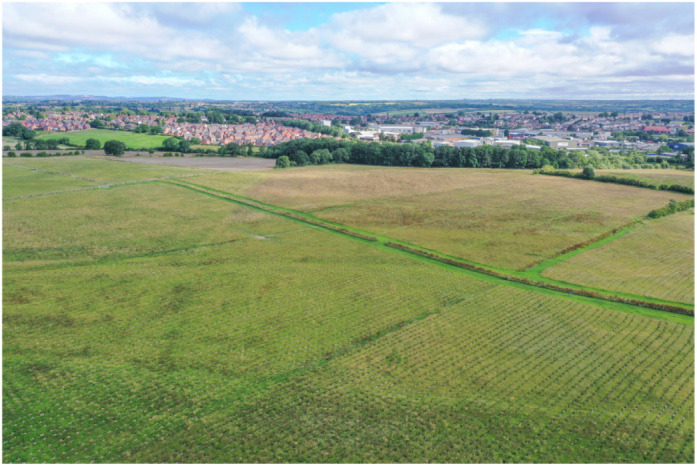
**Trees planted at the Young People's Forest, Derbyshire.** We will be planting a new tree for every research paper (including Resource Articles) and for every peer-reviewed review-type article (e.g. Reviews, Perspectives, Clinical Puzzles, At a Glance poster articles and Special Articles) published in our journals. Image credit: James Reader, Front Row Films/WTML.

## Preserving ancient woodland

Ancient woodlands are some of the rarest and most biodiverse habitats in the UK and home to more threatened species than any other terrestrial habitat in the country. We will be funding the restoration of nearly 12 hectares of degraded temperate rainforest over the next two years in partnership with the Woodland Trust. Great Knott Wood is located on the shore of Lake Windermere within the Lake District National Park. Restoration efforts include the protection of veteran trees and deadwood, removal of non-native species, and the gradual thinning of the canopy to carefully increase light levels. Together, these activities will help counteract biodiversity loss and protect these habitats for future generations.

**Figure:**
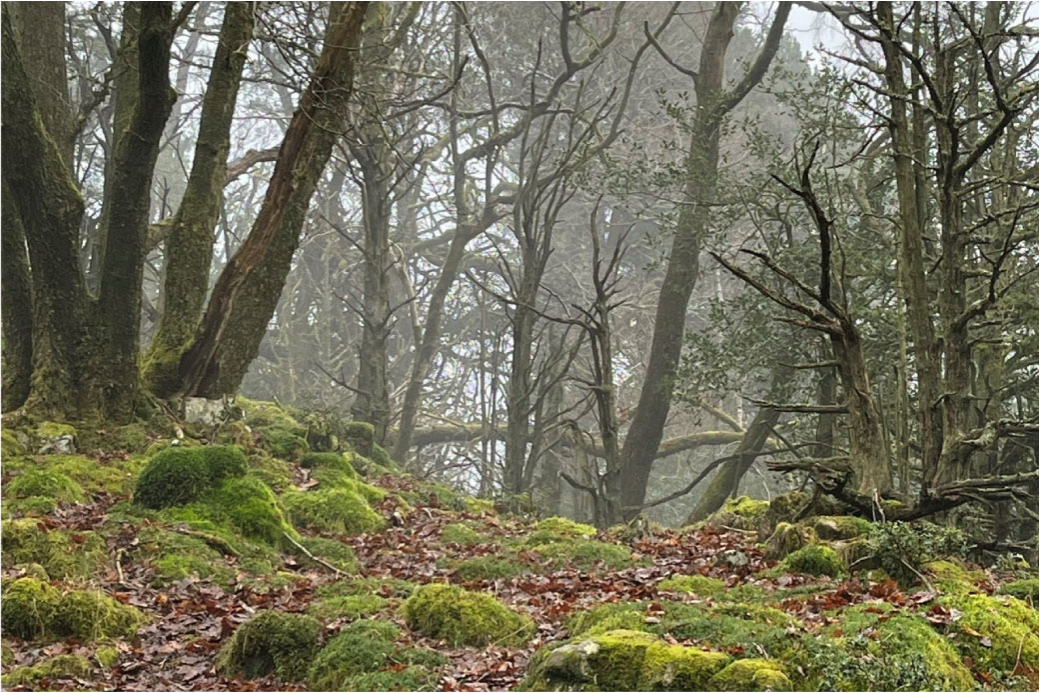
Preservation and restoration of ancient woodland at Great Knott Wood, Lake Windermere.

## Adding a virtual forest

To keep track of our progress, we have created a virtual forest (https://forest.biologists.com) that we hope you'll explore.

For every article published, a new tree is planted – and a representation of that tree will appear in the virtual forest. Authors will be able to see which species has been planted on their behalf (this will match a real tree). Readers will see clickable forest icons for articles published from January 2023 and will be able to browse articles (trees) within the forest.

Similarly, each time a peer reviewer completes the review process for one of our articles, we will dedicate a tree in the ancient woodland to them. Virtual representations of these trees will be added to the forest periodically and there will be no association with specific articles so that peer reviewers retain their anonymity.

*As Editor-in-Chief, I am delighted that DMM is participating in this exciting new initiative. Community is at the heart of what we do, and I am excited to see our forest grow on behalf of our authors and expert reviewers.*

Liz Patton, Editor-in-Chief

**The virtual forest**

Virtual trees will appear in our online forest to represent each new tree planted on behalf of our authors and trees preserved in ancient woodland to acknowledge our peer reviewers. (A) A new ‘leaves’ icon on articles will allow readers to click through to view individual trees. (B) View of the landscape in the virtual forest. (C) Information will be provided about each tree species along with a button to tweet individual trees (#forestofbiologists).

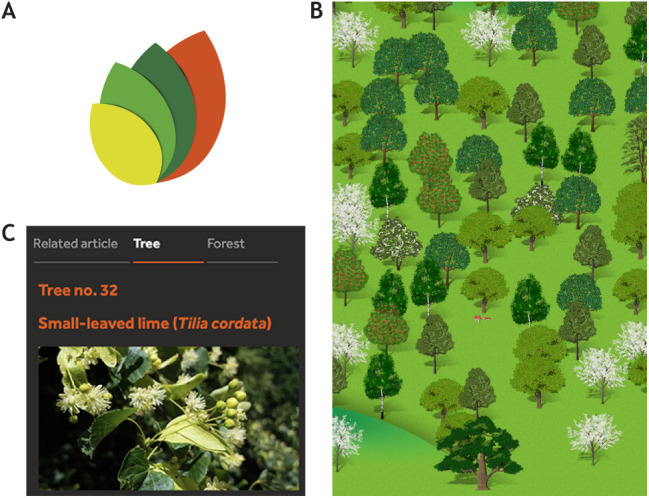

## Growing a sense of community

This project was developed as a community initiative. We would particularly like to thank Steve Kelly, Professor of Plant Sciences, University of Oxford (and Editor-in-Chief of Biology Open), for planting the seed that grew into The Forest of Biologists. The idea was enthusiastically embraced by our Board of Directors who approved the financial commitment needed to ensure that our efforts were appropriate, long term and supported by science.

As we look ahead to the centenary of The Company of Biologists in 2025, we've been thinking deeply about our contributions to biology and the scientific communities around our journals. The Forest of Biologists represents an important step in our commitment to incorporating sustainability thinking into all aspects of what we do (https://www.biologists.com/about-us/sustainability/) as we look forward to another 100 years of supporting biologists and inspiring biology.

